# Real-World Evidence of Prostatic Urethral Lift Confirms Pivotal Clinical Study Results: 2-Year Outcomes of a Retrospective Multicenter Study

**DOI:** 10.1089/end.2019.0167

**Published:** 2019-07-12

**Authors:** Gregg Eure, Steven Gange, Peter Walter, Ansar Khan, Charles Chabert, Thomas Mueller, Paul Cozzi, Manish Patel, Sheldon Freedman, Peter Chin, Steven Ochs, Andrew Hirsh, Michael Trotter, Douglas Grier

**Affiliations:** ^1^ Urology of Virginia, Virginia Beach, Virginia.; ^2^ Summit Urology Group, Salt Lake City, Utah.; ^3^ Western NY Urology Associates, Jamestown, New York.; ^4^ Urology Heath Center, Fremont, Nebraska.; ^5^ The Prostate Clinic, Benowa, Australia.; ^6^ Delaware Valley Urology, Voorhees, New Jersey.; ^7^ Dr. Paul Cozzi, Hurstville, Australia.; ^8^ Advanced Urology & Women's Health Center, Elgin, South Carolina.; ^9^ Sheldon Freedman, MD LTD, Las Vegas, Nevada.; ^10^ South Coast Urology, Wollongong, Australia.; ^11^ Urology One, Canton, Ohio.; ^12^ Jersey Urology Group, Somers, New Jersey.; ^13^ Midtown Urology Associates, Austin, Texas.; ^14^ Sound Urological Associates, Edmonds, Washington.

**Keywords:** lower urinary tract symptoms, LUTS, retrospective study, real world, prostatic urethral lift, PUL, benign prostatic hyperplasia, BPH

## Abstract

***Introduction:*** This study expands results from recent prostatic urethral lift (PUL) clinical trials by examining outcomes within a large unconstrained multicenter data set.

***Methods:*** Retrospective chart review and analysis of 1413 consecutive patients who received PUL in North America and Australia was performed. International Prostate Symptom Score (IPSS), quality of life (QoL), and maximum urinary flow rate (Qmax) were evaluated at 1, 3, 6, 12, and 24 months post-procedure for all nonurinary retention subjects (Group A) and retention subjects (Group B). Within Group A outcomes were further analyzed using paired *t*-tests and 95% mean confidence intervals under the following parameters: IPSS baseline ≥13, age, prostate size, site of service, prostate cancer treatment, and diabetic status. Adverse events, surgical interventions, and catheterization rates were summarized in detail.

***Results:*** Compared with the randomized controlled prosatic urethral lift (L.I.F.T.) study, subjects in this retrospective study were older and less symptomatic. After PUL, mean IPSS for Group A improved significantly from baseline by at least 8.1 points throughout follow-up. No significant differences were observed between Group A and B follow-up symptom scores. Within Group A, subjects with an IPSS baseline ≥13 behaved similarly to L.I.F.T. subjects. Age, prostate volume, site of service, prior cancer treatment, and diabetic status did not significantly affect PUL outcomes. When completed in a clinic office, PUL resulted in less side effects and catheter placement compared to other sites of service. Previous prostate cancer treatment did not elevate adverse events of high concern such as incontinence and infection.

***Conclusion:*** PUL performs well in a real-world setting in terms of symptom relief, morbidity, and patient experience for all studied patient cohorts.

## Introduction

Benign prostatic hyperplasia (BPH) is a chronic age-related condition associated with insidious lower urinary tract symptoms (LUTS) that include urinary frequency, urgency, and nocturia. Half of all men between 50 and 60 years are affected with BPH, and as prevalence increases during each decade of life, it is estimated that 90% of men ≥80 years are afflicted.^1^ As BPH advances, voiding is increasingly obstructed and can significantly impact a patient's quality of life by causing loss of sleep, reduced productivity, impaired sex life, social isolation, and depression.^2^

Historically, first lines of treatment for mild LUTS involve watchful waiting and drug therapy.^3^ When symptoms become intolerable, transurethral resection of the prostate (TURP), the gold standard of surgical intervention, has offered patients effective relief from LUTS since its introduction in the early 1900s.^4^ Despite these management options, high proportions of BPH patients are left underserved. More than 25% of medically managed patients are noncompliant or discontinue their medication because of insufficient relief or side effects such as erectile and ejaculatory dysfunction, fatigue, and dizziness.^5,6^ Of this population, the majority forgo surgery because of lengthy recovery times and a 20% rate of perioperative morbidity.^7^

Prostatic urethral lift (PUL) is a minimally invasive approach for BPH developed to address concerns of the large patient population underserved by traditional treatment. Approved by the FDA in 2013, PUL relies on permanent nonabsorbable sutures that mechanically open the prostate fossa. Treatment can be administered in an office setting under local anesthesia.^8^ Outcomes of PUL have been extensively studied in controlled clinical trials involving >500 patients.^8–13^ The procedure offers patients rapid symptom relief with mean International Prostate Symptom Score (IPSS), quality of life (QoL), and maximum urinary flow rate (Qmax) improvement of 40% to 50% at 1 month.^8,9,11–13^ Results are durable, recently shown to be sustained at 5 years (IPSS 36%, QoL 50%, and Qmax 44%) post-treatment.^13^ Notable clinical advantages also involve a quick recovery time, mild to moderate side effects that resolve by 2 to 4 weeks, and low surgical retreatment rates.^13^

Although randomized clinical trials have provided evidence to assess the safety and efficacy of treatments for BPH, the degree to which these results correlate with real-world outcomes has been sparsely explored. Broadly establishing the effectiveness of any treatment option suggests the need for a performance analysis in an unconstrained clinical setting. Although there are smaller population studies,^14,15^ to our knowledge, this investigation is the first of its kind: an analysis of BPH device technology within a real-world setting.

## Materials and Methods

### Study protocol

A protocol-driven retrospective multicenter study of the PUL procedure was performed across 14 sites in the United States and Australia. Sites were initiated into the study between July 2017 and September 2018. All consecutive subjects from each site who had undergone a PUL procedure after market clearance (U.S. FDA clearance September 24, 2013; Australia TGA approval February 2, 2010) up to the site initiation date were included in the study if the following data were available: (1) a documented baseline IPSS score (≤9 months before PUL) and (2) at least one post-procedure IPSS within 12 months of their treatment date. Urinary retention patients were included but exempt from having baseline symptom scores. Enrollment criteria were assessed under a series of individual chart reviews and no data were included after the site initiation date. Chart reviews at each site were conducted once IRB approval was received. All study procedures were performed in full accordance with all applicable U.S. Federal and state laws and regulations, including 45 CFR 46 and the HIPAA Privacy Rule.

### Study procedures

**FIG. 1.**
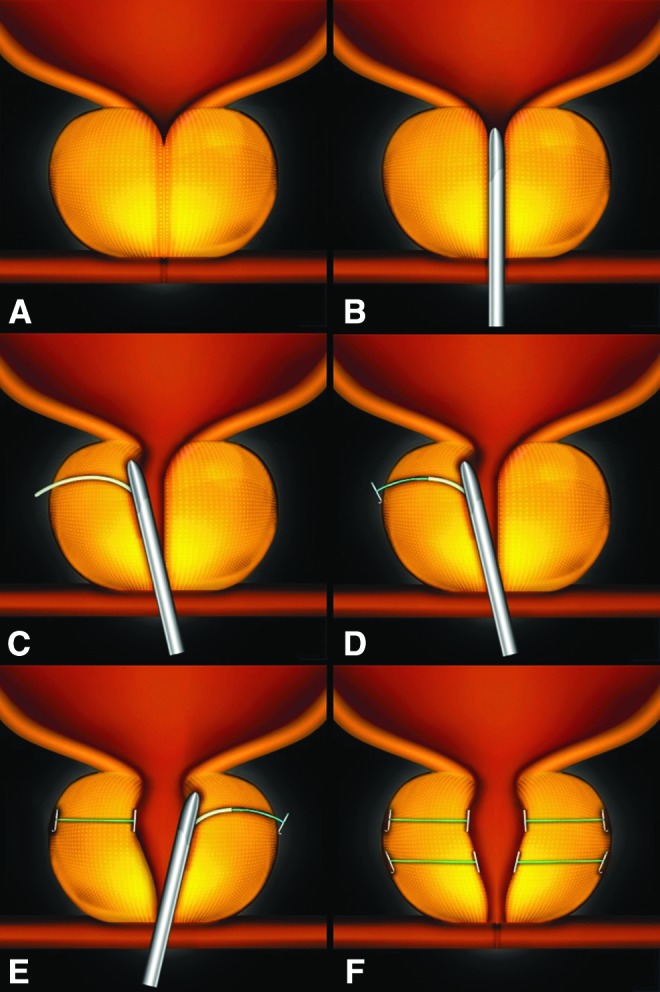
PUL procedure depicting implant delivery sequence and the result effect on a prostate obstructed by BPH. The implant is delivered into the encroaching lateral lobes of a prostate **(A)** by introducing the device under cystoscopic guidance **(B)**, compressing the lobe with the delivery device, deploying a needle through the prostatic lobe and capsule **(C)**, retracting the needle, tightening tension on the monofilament connector, and securing implant with a urethral end-piece **(D)**. Additional implants are delivered as required **(E)** to maintain an expanded urethral lumen **(F)**. BPH, benign prostatic hyperplasia; PUL = prostatic urethral lift. (Images copyrighted and printed with permission by NeoTract, Inc.)

During the PUL procedure, transprostatic implants (UroLift^®^ System; NeoTract, Pleasanton, CA) are placed under endoscopic guidance to mechanically disassociate the obstructing prostatic lobes and expand the urethral lumen. After rigid cystoscopy, the implant delivery device is inserted into a 20F sheath and angled laterally to compress the obstructive lobe. A 19-gauge needle is deployed extending from the intraluminal urethral wall through the prostatic capsular surface. The capsular tab is delivered through the hollow needle, is retracted, and engages the prostatic capsule. The monofilament is then tensioned and secured in place by the urethral end-piece (Fig. 1).

### Study assessments

Chart review was performed for 1423 subjects, 10 of whom were later determined to fail criteria for in-depth analysis. The total study population was, therefore, 1413 analyzed subjects. Baseline demographics and symptom outcomes for the total study population were compared with those reported in the randomized controlled prosatic urethral lift (L.I.F.T.) study.^13^ Group A subjects provided baseline symptom and flow data, whereas Group B subjects were in urinary retention at baseline. Mean differences and percentage change from baseline for IPSS, QoL, and Qmax were analyzed at 1, 3, 6, 12, and 24 months post-PUL using paired *t*-tests and 95% confidence intervals (CIs) for Group A, as well as for Group A cohorts. Subgroup analyses were conducted for moderate to severe symptoms (IPSS ≥13 [*n* = 1047]), age (<50 years [*n* = 17]), prostate volume (<30 cc [*n* = 165]; 30 to <60 cc [*n* = 353]; 60 to <80 [*n* = 105] and ≥80 cc [*n* = 38]), site of service (clinic office, *n* = 392), prior prostate cancer treatment (*n* = 73) and diabetes (*n* = 243). Absolute symptom scores after PUL were statistically compared between Groups A and B. Adverse events, surgical retreatments, and catheterization rates were independently calculated for Groups A and B.

## Results

A total of 1413 subjects, 1248 spontaneously voiding subjects (Group A) and 165 urinary retention subjects (Group B), constituted this real-world retrospective (RWR) study. The average duration of subject follow-up was 273 days. Compared with the L.I.F.T. study, RWR subjects were older (70 *vs* 67 years, *p* < 0.001, Table 1), had lower baseline IPSS (19.2 *vs* 22.3, *p* < 0.0001), lower QoL (4.0 *vs* 4.6, *p* < 0.0001), and higher Qmax (12.6 *vs* 7.9 mL/second, *p* < 0.0001). Seventeen subjects <50 years received PUL and experienced similar symptom improvement compared to older subjects.

**Table 1.** Baseline Demographics of the RWR Total Study Population and L.I.F.T. Study Subjects

*Mean, median, SD, range,* n	*RWR*	*L.I.F.T. (5 year)*	p*-Value*
Age	70, 70, 9.0 [35–96], (1413)	67, 67, 8.6 [49–86], (140)	<0.001
BMI	29, 28, 6.2 [0–86], (1156)	29, 29, 4.6 [19–47], (137)	0.5
Prostate volume (cc)	45, 41, 21 [13–158], (753)	45, 42, 12 [30–77], (140)	0.7
IPSS	19, 19, 6.9 [1.0–35], (1317)	22, 22, 5.5 [13–35], (140)	<0.0001
QoL	4.0, 4.0, 1.6 [0–35], (1134)	4.6, 5.0, 1.1 [2.0–6.0], (140)	<0.0001
Qmax	13, 11, 7.3 [2.0–69], (515)	7.9, 8.0, 2.4 [3.0–13], (140)	<0.0001
PVR	135, 77, 172 [0–1000], (1052)	86, 72, 69 [0–246], (140)	<0.001
PSA	4.1, 1.7, 38 [0–1067], (897)	2.4, 1.9, 2.0 [0.1–11], (140)	0.6
Implants per subject	4.6, 4.0, 1.3 [2.0–10], (1413)	4.9, 4.0, 1.6 [2.0–11], (140)	0.01

BMI = body mass index; IPSS = International Prostate Symptom Score; L.I.F.T. = randomized controlled prostatic urethral lift; Qmax = maximum urinary flow rate; QoL = quality of life; PSA = prostate-specific antigen; PVR = post-void residual; RWR = real-world retrospective.

**FIG. 2.**
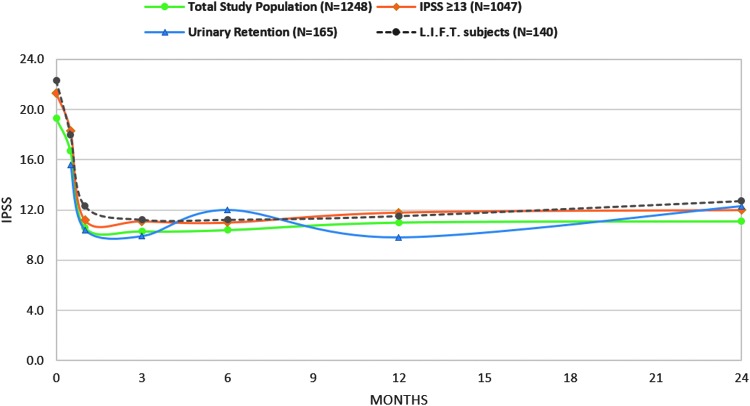
IPSS response to PUL in RWR subjects compared with pivotal clinical study (L.I.F.T.) subjects. IPSS = International Prostate Symptom Score; RWR = real-world retrospective.

After PUL, IPSS values for Group A improved significantly from baseline at all timepoints by at least 8.1 points (Fig. 2 and Table 2, *p* < 0.0001). Mean QoL improved at 24 months by 41% (Table 2). For subjects with baseline IPSS ≥13, IPSS improvement (Fig. 2) and percentage change per timepoint were not significantly different compared with subjects from the L.I.F.T. study at 1, 3, 6, 12, and 24 months. Most perioperative adverse events were mild to moderate and resolved by 4 weeks. Over the course of the study, 72 subjects underwent either a PUL retreatment (*n* = 39) or an alternative surgical intervention (17 laser procedures and 16 TURPs), 11 of which included removal of implants. Only one additional subject required a procedure specifically to remove a UroLift System implant.

**Table 2.** Response to Prostatic Urethral Lift in the RWR Total Study Population and RWR Subjects with International Prostate Symptom Score Baseline ≥13

		*Timepoint*									
		*1 Month*		*3 Months*		*6 Months*		*12 Months*		*24 Months*	
*Test*		*Total cohort*	*IPSS baseline ≥13*	*Total cohort*	*IPSS baseline ≥13*	*Total cohort*	*IPSS baseline ≥13*	*Total cohort*	*IPSS baseline ≥13*	*Total cohort*	*IPSS baseline ≥13*
IPSS	*n* (paired)	795	666	506	427	229	193	241	204	151	131
	Baseline	19.3 ± 6.8	21.3 ± 5.4	19.1 ± 6.7	21.1 ± 5.2	19.1 ± 6.8	21.2 ± 5.2	19.1 ± 7.0	21.0 ± 5.6	19.5 ± 7.2	21.2 ± 5.8
	Change	−8.6	−10.1	−8.7	−9.9	−8.7	−10.2	−8.1	−9.2	−8.3	−9.2
	% Change	−39	−45	−42	−46	−41	−47	−39	−43	−37	−40
	*p*-Value	<0.0001	<0.0001	<0.0001	<0.0001	<0.0001	<0.0001	<0.0001	<0.0001	<0.0001	<0.0001
QoL	*n* (paired)	639	532	430	363	189	164	190	162	118	98
	Baseline	4.0 ± 1.8	4.2 ± 1.8	3.9 ± 1.3	4.1 ± 1.2	4.0 ± 1.2	4.2 ± 1.2	4.0 ± 1.3	4.2 ± 1.2	3.9 ± 1.2	4.2 ± 1.1
	Change	−1.9	−2.1	−1.9	−2.0	−1.8	−1.9	−1.7	−1.8	−1.7	−1.7
	% Change	−43	−46	−45	−46	−43	−44	−39	−40	−41	−39
	*p*-Value	<0.0001	<0.0001	<0.0001	<0.0001	<0.0001	<0.0001	<0.0001	<0.0001	<0.0001	<0.0001
Qmax	*n* (paired)	208	166	148	118	47	42	73	56	30	26
	Baseline	13.2 ± 8.2	12.6 ± 7.1	13.1 ± 7.4	12.6 ± 7.6	13.4 ± 6.3	13.1 ± 6.3	13.9 ± 10.2	13.3 ± 10.6	12.6 ± 4.8	12.3 ± 5.1
	Change	1.3	1.7	2.3	2.3	2.3	2.8	−0.4	0.2	2.1	2.9
	% Change	29	30	31	31	36	41	15	19	32	40
	*p*-Value	0.02	<0.01	<0.01	<0.01	0.03	0.02	0.7	0.85	0.08	0.03

Within Group A, postoperative catheters were placed in 411 subjects as a standard of care at that site. Of the remaining 837 subjects, 704 (84%) required no catheter. The rate of catheter independence, including those placed following standard of care, was 90% at 48 hours, 94% at 5 days, 98% at 1 month, and 99.5% at study end.

Thirty-seven percent of Group B subjects were catheterized >3 months before PUL, 20% between 1 and 3 months, and 43% <30 days. Catheter independence was achieved by 69% of Group B subjects 5 days after PUL, 83% by 1 month, and 87% by the end of the study. Fourteen (14/165) subjects were unable to void spontaneously and underwent an additional surgical intervention by study end. Although baseline symptom scores and uroflowmetry assessments were not available because of retention status, absolute scores for 3- and 12-month follow-up were as follows: 9.9 and 9.8 (IPSS; Fig. 2); 1.7 and 1.9 (QoL); 11.1 and 10.2 mL/second (Qmax).

**FIG. 3.**
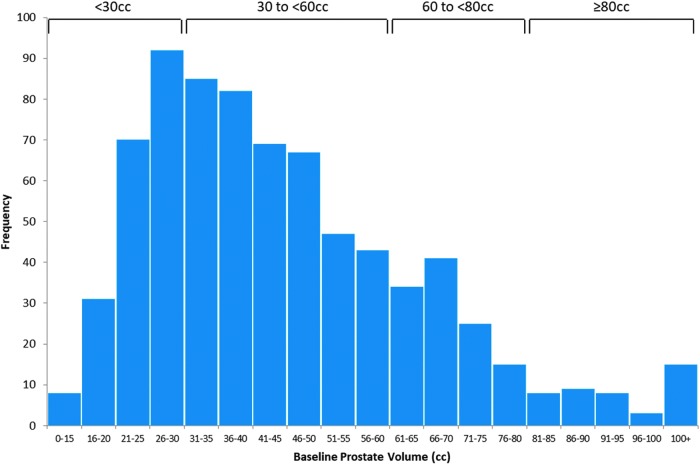
Prostate volume distribution within the RWR total study population.

**FIG. 4.**
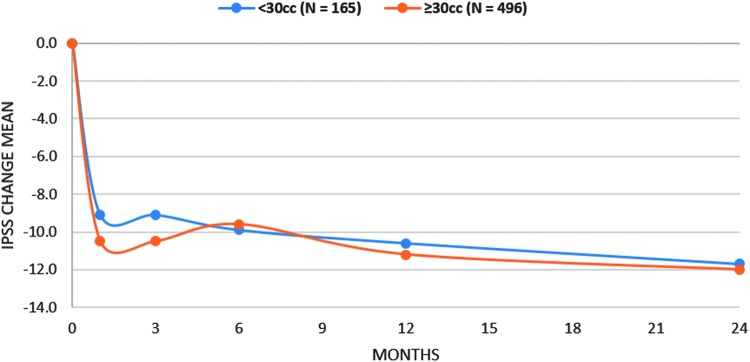
IPSS improvement in RWR subjects with prostate volumes less than or greater than or equal to 30 cc.

Median and mean prostate size (determined predominantly by transrectal ultrasonography) for the RWR total study population was 41 and 45 cc, 95% CI (43.7–46.7), respectively (Fig. 3). Subjects received an average of 4.6 implants (±1.3, range 2–10). No significant differences in symptom response emerged based on prostate volume. Group A subjects with prostate volumes <30 cc (*n* = 165) had significant improvements from baseline at all timepoints and effectiveness in this group was comparable to the subjects with prostate volumes ≥30 cc (*n* = 496; Fig. 4). Although small patient numbers beyond 6 months limited analysis, 38 subjects with prostates ≥80 cc experienced similar absolute symptom scores throughout follow-up compared to subjects with smaller prostates (<80 cc, *n* = 623; IPSS baseline: 19.4 *vs* 17.6, *p* = 0.1; 1 month: 10.6 *vs* 9.0, *p* = 0.3; 6 months: 10.0 *vs* 9.6, *p* = 0.8). There were also no significant differences in overall adverse event rate (*p* = 0.5) and catheter-free rates (*p* = 0.1) after PUL.

In the United States, 1141 PUL procedures were conducted: 46% with general anesthesia, 30% with local anesthesia, and 25% with twilight anesthesia. In Australia, 272 procedures were conducted: 99% of which were performed with general anesthesia in a hospital setting. Across sites in the United States, 39% (446 of 1141) of procedures were conducted in the clinic office, 74% (330/446) of which were completed using only local anesthesia. IPSS outcomes after PUL were not significantly affected by site of service (i.e., clinic office, hospital, or ambulatory surgery center); however, subjects treated in the office did experience fewer perioperative adverse events (Table 3) and catheters (*p* < 0.01). Significant improvements in perioperative outcomes were also found when comparing local *vs* higher levels of anesthesia (intravenous ± laryngeal mask airway) in the clinic office (adverse event rate 24% *vs* 38%, *p* < 0.01 and catheter-free rate 81% *vs* 7%, *p* < 0.0001).

**Table 3.** New Onset Adverse Events with Significant Differences in Occurrence Rate Between Clinic Office and Other Sites of Service

*Type*	*Clinic office subjects (*n* = 392)*		*All other subjects (*n* = 854)*		p*-Value*
	*No. of events*	*No. of subjects (%)*	*No. of events*	*No. of subjects (%)*	
Any adverse event	151	100 (25.5)	596	353 (41.3)	<0.0001
Hematuria	49	43 (11.0)	195	176 (20.6)	<0.0001
Dysuria	8	8 (2.0)	77	75 (8.8)	<0.0001
Incontinence^a^	3	3 (0.8)	28	28 (3.3)	<0.01
Pelvic pain	2	2 (0.5)	23	21 (2.5)	0.02
Urinary urgency	2	2 (0.5)	41	40 (4.7)	<0.0001
Urinary frequency	1	1 (0.3)	15	15 (1.8)	0.03

^a^ Data capture did not allow for incontinence differentiation.

**FIG. 5.**
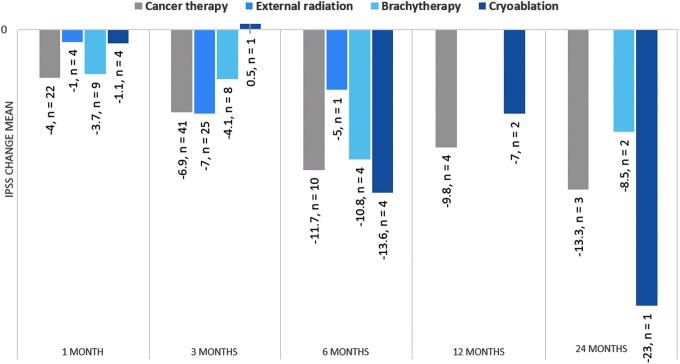
IPSS improvement for RWR prostate cancer therapy subjects in response to PUL.

Medical history revealed 108 RWR subjects with a history of prostate cancer (CaP), 73 of whom received the following cancer therapy: external radiation (*n* = 28), brachytherapy (*n* = 17), cryoablation (*n* = 10), and androgen deprivation therapy ± chemotherapy (*n* = 18). Baseline IPSS (18.6), QoL (4.1), and Qmax (11.4) for CaP therapy subjects did not differ from the RWR total study population, and the average duration from cancer diagnosis to PUL was 4.6 years (range 0.8–196 months). After PUL, mean IPSS for CaP therapy subjects improved at all timepoints (range 4–13.3; Fig. 5) and subanalysis revealed symptom relief across all cancer therapy cohorts. CaP therapy subjects did not experience any serious bleeding or painful urination events or significant increases in incontinence (*p* = 0.09), urinary tract infection (*p* = 0.8), urosepsis (*p* = 1.0), or urethral stricture (*p* = 0.05) compared to subjects without cancer.

After PUL, mean IPSS and QoL for Group A diabetic subjects (*n* = 243) improved significantly from baseline at all timepoints (*p* < 0.001). In addition, univariate regression analysis of baseline body mass index (BMI) revealed that BMI did not significantly impact PUL IPSS improvement.

## Discussion

This study elucidates how PUL has performed beyond the scope of highly controlled clinical trials and provides new insights on effectiveness, safety and postprocedural standard of care within previously unexamined patient populations suffering from LUTS/BPH.

Patients in this RWR study were modestly older and less symptomatic compared with subjects in the randomized L.I.F.T. study.^13^ In the real world, it may be that men who are earlier in their disease process are unhappy with medications and seek an interventional solution. Recent recommendations by the AUA suggest treating patients during early stages of disease may preclude the progression of bladder dysfunction and retention.^16^ Prostates <30 cc responded well to PUL and constituted a notable proportion of patients within this retrospective study (Fig. 2). Unlike 5-ARI treatment and some thermal therapies that are not indicated for the treatment of prostates <30 cc, PUL has no lower bound on prostate volume in its indication. Far fewer BPH patients have prostates >80 cc, but it is encouraging to see that PUL was similarly effective in this group as well. The modestly attenuated symptom improvement in this study compared with L.I.F.T. is expected due to the known effect of baseline symptom score on subsequent symptom improvement.^17,18^ When analyzing RWR subjects matched to L.I.F.T. baseline criteria (IPSS ≥13), differences in symptom improvement disappear. Rates of adverse events for RWR subjects are also comparable with previous controlled studies.^19,20^ In the L.I.F.T. study, 10.7% (15/140) of subjects required a procedure to remove implants to rectify placement issues. As a result, deployment accuracy became a central issue in technique training. It is encouraging that considerably fewer subjects in this real-world retrospective study required implant removal.

Upon FDA approval in 2013, PUL was indicated for the treatment of prostates <80 g in men ≥50 years. The robust symptom improvements seen within RWR subjects <50 years and prostate sizes <30 and >80 provides evidence that PUL can significantly benefit these patients. PUL has been shown in clinical studies to be tolerated under local anesthesia in an office setting with no need for postoperative catheter in 80% of subjects.^8^ This study corroborated this (84% with no catheter in void trial tested patients) although it appears that many men still endure a postoperative catheter after PUL simply because of facility/urologist preference. Our results show that in a real-world setting, PUL treatment in a clinic office may be associated with better outcomes. Compared to treatments conducted in other settings, office-based procedures were associated with similar symptom improvements with fewer adverse events and less frequent catheterization. These findings were also seen for office procedures performed with local anesthesia compared to higher levels of anesthesia and may be a result of the standard of care at treating facilities, attentive monitoring of patients under local anesthesia, less tendency to overdistend the bladder, and sensitivity to reducing urethral trauma in the local anesthetic office-based setting.

RWR retention patients, a cohort not previously well studied, demonstrate similar absolute symptom scores after PUL compared to nonurinary retention subjects. By study end, 87% of retention subjects were catheter free. A similar rate of catheter independence at 24 months (86%) for a small population of retention patients treated with PUL (*n* = 14) was reported in 2018.^14^ PUL success in retention patients compares well with the 88% success rate reported after TURP.^7^

Prostate cancer frequently coexists with BPH^21^ and may require treatment with radiation or cryoablation. These modalities are often accompanied by hematuria,^22,23^ dysuria,^22^ urinary stricture,^23^ and incontinence^22,24^ lasting months to years after treatment. The real-world retrospective study provided an opportunity to perform analysis on subjects who received radiation or cryoablation before PUL. Subgroup analysis revealed symptom relief across all cancer therapy cohorts without increasing postoperative adverse events of interest (strictures, incontinence, infection, sepsis, and serious bleeding). Conversely, increased rates (between 18% and 70%) of stress incontinence have been documented after TURP in postradiation prostate cancer patients.^25,26^ This preliminary analysis suggests PUL can provide safe symptom relief to patients treated for prostate cancer also suffering from bothersome LUTS.

Recently, a retrospective study, including >9000 male diabetic and nondiabetic patients taking BPH medication, revealed that men with diabetes experienced more bothersome LUTS and reduced urine flow compared to their nondiabetic counterparts.^27^ Within this study, we found no IPSS, QoL, or Qmax baseline differences between diabetic and nondiabetic subjects, and demonstrated that PUL can equally improve symptoms.

The strengths and weaknesses of this retrospective real-world retrospective study have prompted the development of a protocol for an imminent prospective multicenter study. An important weakness of electronic medical record data is unreliable reporting of medication usage, particularly with regard to discontinuation of chronic prescriptions, which typically need to be manually entered. This weakness of EMR systems has been commonly described^28^ and the retrospective IRB protocol did not allow for prospectively contacting subjects to verify records or gain more detail on clinical outcomes. In addition, instrumentation data such as Qmax machine calculations are known to be erroneous due to artifact but no manual over-reading of waveforms was possible, which may at least partially account for the somewhat muted Qmax response to PUL. Another limitation of the study was that the anesthetic regimens at each site were not captured sufficiently to allow for detailed comparisons in approach based on the differences in use of oral sedation, prostate block, and anesthetic gel. Finally, sexual function, which can be an important impetus behind selecting PUL treatment, was rarely formally reported as part of the standard protocol per institution and thereby limited our ability to make assessments.

## Conclusion

This study advances the field by providing a large data set reflective of the real-world BPH patient from which to make evidence-based recommendations. These results indicate that patients outside the controlled setting of a clinical trial and with baseline characteristics not previously studied (i.e., prostate volume <30 cc, prostate volume >80 cc, moderate IPSS symptoms <13, and history of prostate cancer) can be treated safely and effectively with the PUL procedure. PUL appears to perform well as a routine outpatient procedure in terms of symptom relief, catheter requirement, and perioperative morbidity for all analyzed patient cohorts.

## Abbreviations Used

BPH: benign prostatic hyperplasia
CaP: cancer of the prostate
IPSS: International Prostate Symptom Score
L.I.F.T.: randomized controlled prostatic urethral lift
LUTS: lower urinary tract symptoms
PUL: prostatic urethral lift
Qmax: maximum urinary flow rate
QoL: quality of life
RWR: real-world retrospective
TURP: transurethral resection of the prostate
